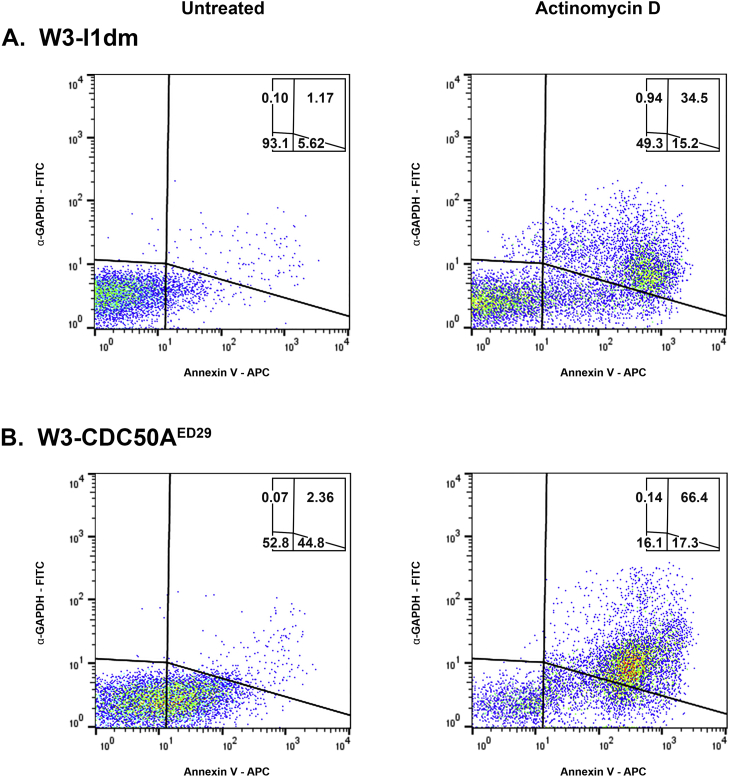# Phosphatidylserine externalization by apoptotic cells is dispensable for specific recognition leading to innate apoptotic immune responses

**DOI:** 10.1016/j.jbc.2022.102034

**Published:** 2022-05-16

**Authors:** Marta T. Gomes, Karol Palasiewicz, Varsha Gadiyar, Kevin Lahey, David Calianese, Raymond B. Birge, David S. Ucker

**Affiliations:** 1 Department of Microbiology and Immunology, University of Illinois College of Medicine, Chicago, Illinois, USA; 2 Department of Microbiology, Biochemistry and Molecular Genetics, Rutgers-New Jersey Medical School, Newark, New Jersey, USA

**Keywords:** apoptosis, immunomodulation, membrane determinants, phosphatidylserine, glycolytic enzyme molecules, caspase-dependence, phagocytosis, cytofluorimetry, APC, allophycocyanin, CFDA, 5(6)-carboxyfluorescein diacetate N-succinimidyl ester, DAMPs, damage-associated molecular patterns, IAI, innate apoptotic immunity, LPS, lipopolysaccharide, PB, Pacific blue, Q-VD-OPh, quinolylvalyl-aspartyl-difluorophenoxy methyl ketone, SUPER, apoptotic determinants localized to the cell surface, ubiquitously-expressed, protease-sensitive, evolutionarily conserved, resident in viable cells, T:R, target cell, responder cell ratio, TAM, Tyro3 / Axl / Mer family, TGFβ, transforming growth factor-β, TLR4, toll-like receptor 4, TMRM, tetramethylrhodamine methyl ester

## Abstract

Surface determinants newly expressed by apoptotic cells that are involved in triggering potent immunosuppressive responses, referred to as “innate apoptotic immunity (IAI)” have not been characterized fully. It is widely assumed, often implicitly, that phosphatidylserine, a phospholipid normally cloistered in the inner leaflet of cells and externalized specifically during apoptosis, is involved in triggering IAI, just as it plays an essential role in the phagocytic recognition of apoptotic cells. It is notable, however, that the triggering of IAI in responder cells is not dependent on the engulfment of apoptotic cells by those responders. Contact between the responder and the apoptotic target, on the other hand, is necessary to elicit IAI. Previously, we demonstrated that exposure of protease-sensitive determinants on the apoptotic cell surface are essential for initiating IAI responses; exposed glycolytic enzyme molecules were implicated in particular. Here, we report our analysis of the involvement of externalized phosphatidylserine in triggering IAI. To analyze the role of phosphatidylserine, we employed a panel of target cells that either externalized phosphatidylserine constitutively, independently of apoptosis, or did not, as well as their WT parental cells that externalized the phospholipid in an apoptosis-dependent manner. We found that the externalization of phosphatidylserine, which can be fully uncoupled from apoptosis, is neither sufficient nor necessary to trigger the profound immunomodulatory effects of IAI. These results reinforce the view that apoptotic immunomodulation and phagocytosis are dissociable and further underscore the significance of protein determinants localized to the cell surface during apoptosis in triggering innate apoptotic immunity.

The physiological consequences of apoptotic cell death are the elimination (clearance) of the dying cell and immune modulation in its environment. The immunomodulatory potency of apoptotic cells, acquired in a caspase-dependent manner as cells die (1), is profound. Apoptotic immune modulation (“innate apoptotic immunity,” IAI) exerts local and even systemic effects. The ability of apoptotic corpses to be recognized by virtually all cell types, notably including macrophages, in a species-independent manner and to modulate the inflammatory responses of those cells (2, 3, 4), is a gain of function acquired paradoxically during the physiological cell death process (5, 6, 7). The ubiquitous recognition of apoptotic cells represents an unconventional immune discrimination. Apoptotic cells target the transcriptional machinery of cells with which they interact, independent of proximal steps of signaling by toll-like receptors (TLRs) and other classical innate immune pattern-recognition receptors. The modulatory activity exerted by apoptotic corpses at all stages of apoptosis manifest as an immediate-early inhibition of proinflammatory cytokine gene transcription and induction of anti-inflammatory cytokine gene transcription and is exerted directly upon recognition, independent of subsequent corpse engulfment or soluble factor involvement (5, 6, 7).

Apoptosis, the primary mode of cell death physiologically (see (8, 9)), is ongoing and extensive throughout metazoan life (in development and homeostasis, even independent of “extra” cell deaths due to pathogen-triggered and other immune functions). While intriguing approaches with therapeutic appeal, exploiting varying aspects of the death process including endoplasmic reticulum and oxidative stresses and mitochondrial outer membrane permeabilization, convert apoptotic cell death to a form that is immunologically provocative (“immunogenic cell death” (10, 11)), apoptotic cell death is primarily and fundamentally immunosuppressive (5, 6, 12, 13, 14). It is likely that most “normal” immune responses *in vivo* occur in a background of calming apoptotic immunosuppression (7). Further, apoptotic immunosuppression is exploited by in tumorigenesis and pathogenesis (13, 14, 15). In those contexts, other modes of regulated cell death, which do trigger active inflammatory and immunological outcomes, also are involved. It is interesting that immunosuppressive apoptosis follows transient waves of proinflammatory cell death, presumably facilitating resolution and wound healing (14, 16).

Membrane lipid asymmetry, including sequestration of negatively charged phospholipids to the inner leaflet of metazoan plasma bilayer, is a common feature of biological membranes (17, 18, 19). Beyond serving as a convenient marker of apoptotic cell death, externalization of the negatively charged phospholipid phosphatidylserine (often with an oxidizable polyunsaturated fatty acid at *sn2* position) plays an essential function in apoptotic cell clearance (20, 21). While it has been assumed that externalized phosphatidylserine serves a similarly essential role in triggering the characteristic immune responses elicited by apoptotic cells, especially *via* engulfment-associated transforming growth factor-β (TGFβ) production (3, 22) or immune signaling following engagement by the Tyro3/Axl/Mer (TAM) family of receptor tyrosine kinases (see later), this view has not been tested rigorously.

Apoptosis-associated externalization of phosphatidylserine is a concerted, caspase-dependent process. Two distinct activities are targeted. One is the caspase-dependent loss of phospholipid flippase (ATP-dependent transport from outer to inner leaflet of the plasma membrane) activity (comprised of a P-type ATPase and its membrane-targeting chaperone, encoded by *ATP11C* and *CDC50A*, respectively; (23, 24, 25)). The second is the caspase-dependent activation of XK-related phospholipid scramblase (bidirectional transport between outer to inner plasma membrane leaflets) activity (26) encoded by the *Xkr8* gene (27).

Noninflammatory clearance of apoptotic cells is accomplished primarily by professional, including tissue resident, phagocytes (2, 3, 4). The ability to ingest apoptotic cells is not restricted to professional phagocytes, however. The clearance of dying cells *in vivo* has been shown to rely in many cases on their engulfment by neighboring homotypic cells (28, 29); this likely is of particular importance in the homeostasis of tissues that maintain barrier functions. Noninflammatory engulfment of apoptotic cells by epithelial cells also has been characterized in a variety of tissues (30, 31, 32, 33). The role of epithelial-derived cells in phagocytosis of apoptotic targets is perhaps best characterized in the eye, where the retinal pigment epithelium is responsible for engulfment (34). One form of retinal dystrophy is a consequence of impaired phagocytosis by retinal pigment epithelium, due to a genetic deficiency of the Mer (Mertk) protein kinase, a member of the TAM family that is essential for engulfment (35).

Phagocytosis-associated signaling arising from TAM activation in dendritic cells has been shown to contribute to the persistence of the immunosuppressive state triggered by apoptotic targets (36). The requirement for *de novo* gene expression, leading to synthesis of SOCS1/3 (among other) proteins, following TAM engagement identifies this as a secondary mechanism of signaling, which may be operative in macrophages and other cells as well. Confoundingly, TAM receptors also have been reported to act independently of engulfment (37, 38) and in those cases, they appear to act in a manner that can be characterized as proinflammatory rather than anti-inflammatory.

Critical evaluation of the assumption that clearance of the apoptotic corpse is itself the trigger for immune modulation and inflammatory suppression (3, 22, 39, 40, 41, 42) is lacking. Although defective (diminished or delayed) phagocytic clearance of apoptotic cells is associated with instances of autoimmune and inflammatory pathology (*e.g.*, systemic lupus erythematosus (40, 43)), the basis of pathologies associated with delayed clearance is not established. Genetic ablation studies (44, 45, 46, 47) do not demonstrate a direct link between the inflammatory state and phagocytosis *per se*, as distinct from other signaling functions associated with the targeted gene products, as we have noted previously (48, 49). Evidence suggestive of a causal role for phagocytosis in inflammatory resolution has been obtained only in few cases. For example, in atherosclerosis, the engulfment of apoptotic cells dependent on activation of the protein tyrosine kinase Mer is required for the production of resolving cytokines and lipid mediators (50).

A molecular focus on proinflammatory determinants recognized by innate immune pattern recognition receptors (especially death-related determinants, so called “damage-associated molecular patterns” [DAMPs]) has led to the notion that it is the overt or cryptic presentation of these apoptotic mimics of pathogenic determinants (pathogen-associated molecular patterns) that is decisive (see (40, 51)). A role for caspases in the destruction of DAMPs has been suggested (51, 52). Remarkably, however, caspase activity has been shown to be responsible for the immunogenicity of dead cells in the case of immunogenic cell death (53). In any case, apoptotic immunosuppression acts in a dominant manner to override the responsiveness triggered by pathogen-associated molecular patterns and DAMPs (5, 6, 15, 48, 54, 55), indicating that the absence of DAMPs cannot be determinative of IAI (7).

We have tested the widespread assumption that apoptotic immunomodulation is linked to phosphatidylserine externalization and the phagocytic engulfment of the apoptotic corpse. We now report our analysis *in vitro* of the involvement of externalized phosphatidylserine, employing a collection of cells that do or do not externalize phosphatidylserine constitutively, independently of apoptosis, and their WT parental cells that externalize that phospholipid in an apoptosis-dependent manner. Here, we demonstrate that phosphatidylserine externalization leading to phagocytosis is dissociable from IAI. Indeed, these two aspects of apoptotic cell fate appear to be attributable to entirely distinct apoptotic cell surface determinants.

## Results

### Dissociation of phosphatidylserine externalization and caspase-dependent apoptosis

We characterized a collection of cell lines that do or do not externalize phosphatidylserine constitutively, independently of apoptosis, together with their WT parental lines that do externalize phosphatidylserine in a typical, apoptosis-dependent manner. The externalization of phosphatidylserine was assessed here simply by the cytofluorimetric detection of binding of fluorescently labeled annexin V.

**Figure 1:**
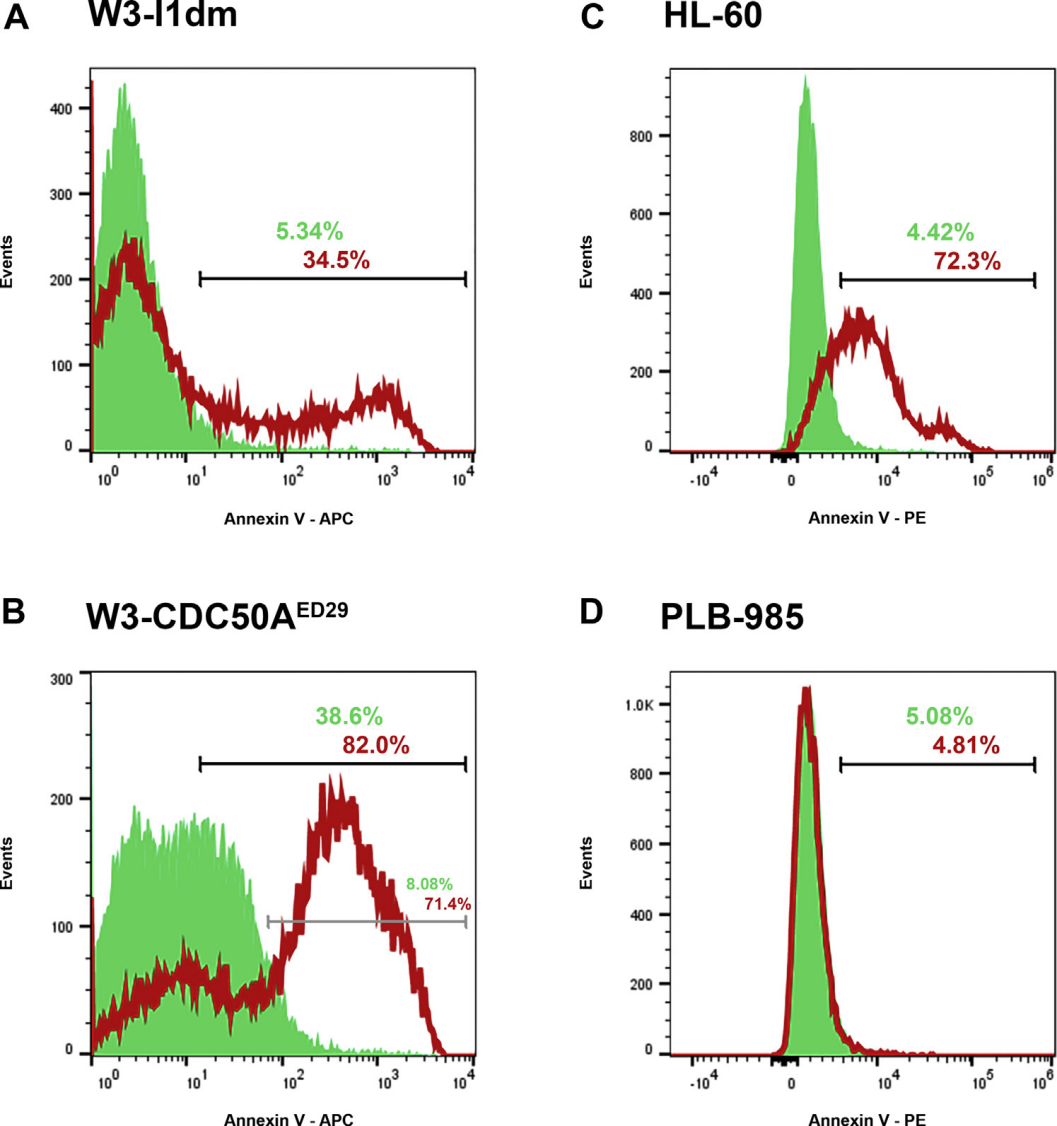
**The externalization of phosphatidylserine is uncoupled from apoptotic death.** The externalization of phosphatidylserine by viable cells (not treated to undergo apoptosis; *green solid histograms*) and by cells treated to undergo apoptosis (*red histogram outlines*) was assessed. Phosphatidylserine externalization was detected cytofluorimetrically by staining with annexin V coupled to APC (W3 - I1dm [*A*] and W3 - CDC50A^ED29^ [*B*] cells) or PE (HL-60 [*C*] and PLB-985 [*D*] cells). Cell death was induced in W3 - I1dm (*A*) and W3 - CDC50A^ED29^ (*B*) cells with actinomycin D and in HL-60 (*C*) and PLB-985 (*D*) cells with staurosporine. Cells that had lost plasma membrane integrity (PI^+^, low forward- and side-angle light scatter) were excluded from these analyses by electronic gating. The fractions of treated and untreated PI^-^ cells exposing substantial (*A*, *B*, *C*, and *D*) and elevated (*B*; see text) levels of phosphatidylserine are indicated (in *green* or *red* type, respectively) above the region markers. Cytometric data of PLB-985 (*D*) and its parent HL-60 (*C*) are displayed on biexponential axes to enhance representation of externalized phosphatidylserine. Panels (*A*) and (*B*) and panels (*C*) and (*D*) present data from single experiments, which are representative of more than 10 independent experiments. APC, allophycocyanin; PI, propidium iodide.

As shown in Figure 1 panels (*A*) and (*B*), while a normal murine T cell line, W3 - I1dm, externalized phosphatidylserine substantially only as it died (panel *A*; in this case, apoptosis was triggered by treatment with actinomycin D), a derivative cell line, W3 - CDC50A^ED29^, lacking activity of the P-type ATPase phospholipid flippase ATP11C due to the targeted ablation of its chaperone component, CDC50A, constitutively externalized phosphatidylserine (panel *B*). The further externalization of phosphatidylserine upon the induction of apoptosis by W3 - CDC50A^ED29^ cells, above the constitutive level of phosphatidylserine exposure resulting from the absence of ATP11C-specific flippase activity, is consistent with a previous characterization of that cell line (23). This elevated phosphatidylserine externalization (see panel *B*) may reflect the caspase-dependent activation of XK-related scramblase activity (56).

In a complementary case shown in Figure 1 panels (*C*) and (*D*), a human promyelocytic leukemia cell line, PLB-985, failed to externalize phosphatidylserine during apoptosis (panel *D*; in this case, apoptosis was triggered by treatment with staurosporine), while HL-60, the acute promyelocytic leukemia cell line that is the parental source of PLB-985 (57), externalized phosphatidylserine significantly as it died (panel *C*). The lack of *Xkr8* expression in PLB-985 cells accounts for the absence of apoptosis-associated phosphatidylserine externalization of those cells (27).

**Figure 2:**
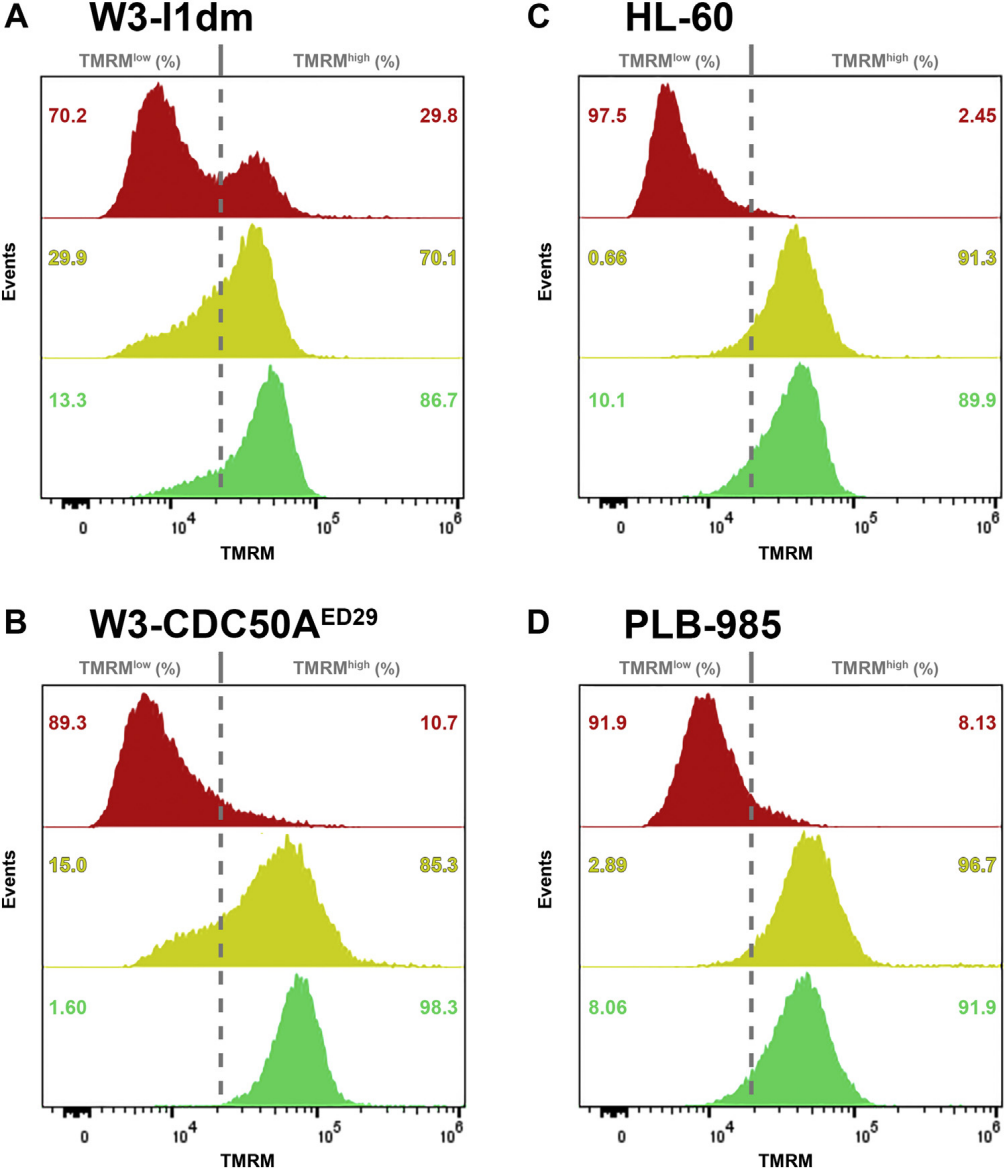
**Cell death uncoupled from phosphatidylserine externalization is caspase dependent.** Cell death was induced in W3 - I1dm (*A*) and W3 - CDC50A^ED29^ (*B*) cells with actinomycin D and in HL-60 (*C*) and PLB-985 (*D*) cells with staurosporine, as in Figure 1, and caspase-dependent apoptosis was inhibited with the pan-caspase inhibitor quinolylvalyl-aspartyl-difluorophenoxy methyl ketone (Q-VD-OPh; 10 μM). Cell death was assessed cytofluorimetrically by the loss of mitochondrial membrane potential, as indicated by the relative intracellular accumulation of tetramethylrhodamine methyl ester (TMRM; (58)) in cells that retained plasma membrane integrity (*i.e.*, in PI^-^ cells with normal forward- and side-angle light scatter). Histograms represent populations of cells treated with those apoptosis-inducing drugs (*red solid histograms*), additionally treated with Q-VD-OPh (*yellow solid histograms*), and untreated controls (*green solid histograms*). The demarcation of regions representing cells that retained mitochondrial membrane potential (TMRM^high^) and that had lost mitochondrial membrane potential (TMRM^low^) is indicated by *dashed vertical lines*, and the fraction of cells within each region for each sample is noted. The figure presents data from a single experiment, which is representative of three independent experiments. PI, propidium iodide.

We have shown previously that the acquisition of immunomodulatory activity specifically by apoptotic cells is dependent on caspase activity (1, 5). We tested that the cell death triggered by actinomycin D and staurosporine, regardless of altered patterns of phosphatidylserine externalization, was, indeed, caspase-dependent apoptosis. Cell death was assessed cytofluorimetrically, by scatter properties and the loss of mitochondrial membrane potential (58). Intracellular (espeacially, mitochondrial) accumulation of the potentiometric dye tetramethylrhodamine methyl ester (TMRM) is diminished as cells lose mitochondrial membrane potential, providing a ready indication of death. As shown in Figure 2, apoptotic death ensued in these cells, unaffected by their phosphatidylserine externalization phenotypes, and inhibited with the pan-caspase inhibitor quinolylvalyl-aspartyl-difluorophenoxy methyl ketone (Q-VD-OPh). In the particular experiment represented in Figure 2, the extent of cell death induced by actinomycin D in W3 - I1dm and W3 - CDC50A^ED29^ cells, as well as its blockade by Q-VD-OPh, were less extensive than the extent of Q-VD-OPh-inhibitable death induced by staurosporine in HL-60 and PLB-985 cells.

Together, these data reinforce the notion that, while phosphatidylserine externalization typically is associated with apoptotic cell death, phosphatidylserine externalization is not essential for—and can be uncoupled from—apoptotic cell death.

### Phosphatidylserine externalized independently of apoptosis functions to trigger recognition and signaling

We examined whether surface-exposed phosphatidylserine externalized independently of apoptosis was able to be recognized and trigger recognition-dependent signaling events comparable to phosphatidylserine externalized during apoptosis. Phosphatidylserine externalization during apoptosis is the predominant determinant for triggering apoptotic cell clearance (20, 21). The initial characterization of W3 - CDC50A^ED29^ cells (23) already demonstrated that constitutive, nonapoptotic externalization of phosphatidylserine resulting from ablation the ATP11C phospholipid flippase resulted in the triggering of phagocytosis. We confirmed this finding (see later and Fig. 6). We tested whether, independent of apoptosis, externalized phosphatidylserine also can trigger other signaling events. For example, receptors of the TAM tyrosine kinase family are known to initiate particular signaling responses following phosphatidylserine engagement (59), dependent on bridging molecules such as growth arrest–specific gene 6 (Gas6) or Protein S (60). The Gas6-dependent activation of Mer appears to exhibit the greatest sensitivity for phosphatidylserine of the TAM receptors (61).

**Figure 3:**
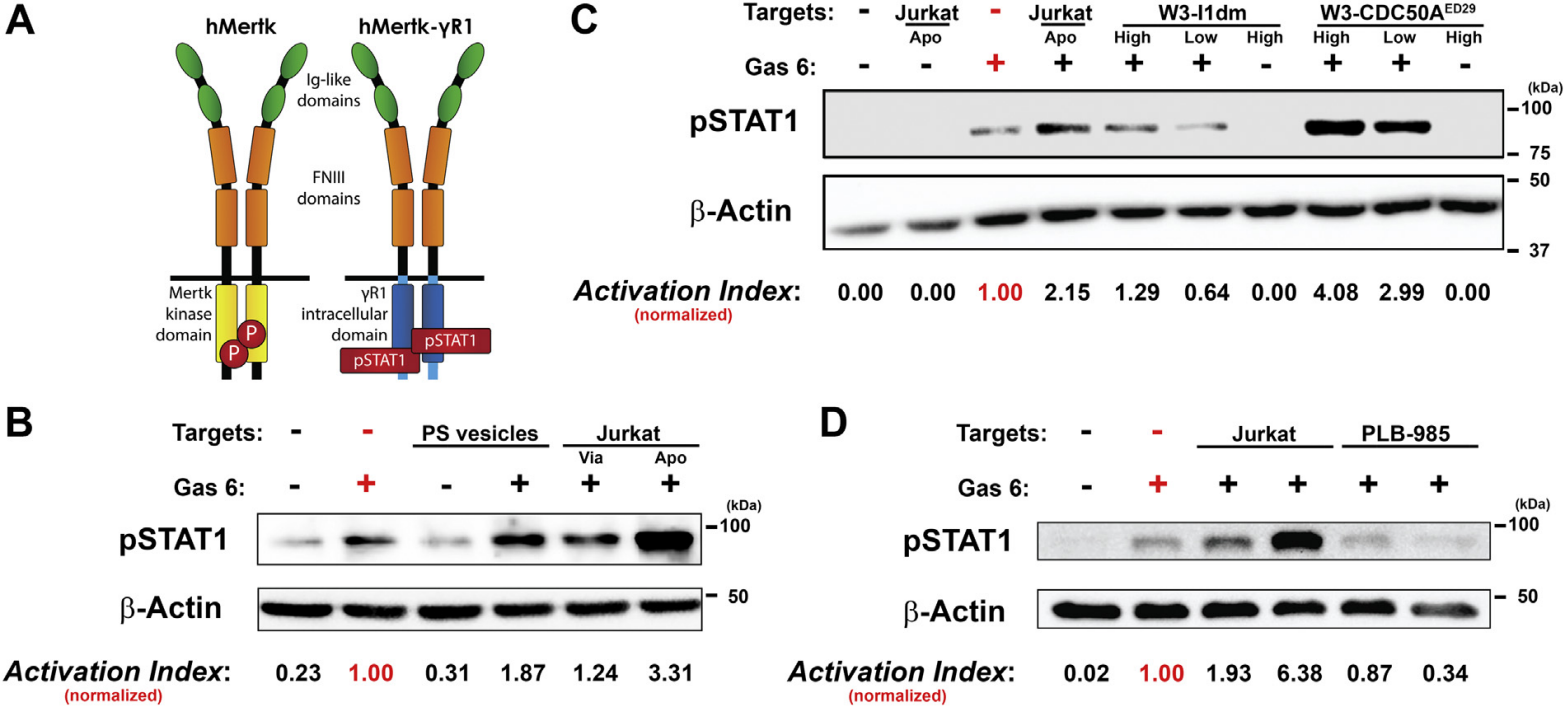
**Phosphatidylserine-dependent signaling responses can be uncoupled from apoptotic cell death.***A*, schematic representation of human Mertk (hMertk) and the hMertk-γR1 reporter construct, composed of the extracellular domain of hMertk and the transmembrane and intracellular domains of IFNγR1 receptor (61). Ligand-induced activation by Gas6 and phosphatidylserine (PS) leads to the phosphorylation of STAT1, which can be assessed by immunoblot analysis. *B*, the reliability of a stable hMertk-γR1 reporter cell line was confirmed using PS vesicles (at a phospholipid concentration of 200 nM), exogenous Gas6, and/or viable or apoptotic Jurkat T cells (at a dose of 1.0 × 10^5^ cells; apoptosis was induced by treatment with staurosporine) as targets. Quantification was accomplished by comparing the densitometric ratios of the phospho-STAT1 (pSTAT1) and control β-actin bands. Determinations were normalized to the value obtained by treatment of reporter cells with Gas6 alone (in *red*) to generate an “Activation Index”. *C*, similarly, the extents of externalization of PS by viable cells of the normal murine T cell line, W3 - I1dm, and by viable cells of the derivative cell line lacking ATP11C phospholipid flippase activity, W3 - CDC50A^ED29^, were assessed functionally at two doses of these smaller target cells (5.0 × 10^6^ cells [“High”] and 1.0 × 10^6^ cells [“*Low*”]). These determinations were compared with those of reporter cells treated with apoptotic (“Apo”) Jurkat T cells induced to die by treatment with staurosporine (1.0 × 10^5^ cells). *D*, the extents of externalization of PS by viable (“*Via*”) and apoptotic (“Apo”) PLB-985 cells as well as viable and apoptotic Jurkat cells (all at a dose of 1.0 × 10^5^ cells) also were assessed functionally. Again, apoptosis was induced in both cell lines by treatment with staurosporine. Experiments shown in panels (*B*), (*C*), and (*D*) were each repeated at least three times, with similar results.

The functionality of externalized phosphatidylserine was assessed with a Mer-derived reporter assay for phosphatidylserine-dependent signaling. The Mer reporter construct is a chimeric protein comprised of the human Mer ectodomain fused to the interferon type I receptor (IFNγR1) transmembrane and cytoplasmic regions (Fig. 3 panel *A*), and it is stably expressed in Chinese hamster ovary cells (61). Activation of the reporter construct, dependent on binding by phosphatidylserine opsonized by γ-carboxylated Gas6, leads to STAT1 phosphorylation (61), providing a sensitive and tractable readout. We confirmed the reliability of the Mer reporter assay with target Jurkat cells, which have been shown previously to externalize phosphatidylserine when induced to die apoptotically (61). As shown in Figure 3*B*, the reporter assay faithfully reports the presence of exposed phosphatidylserine strictly dependent on the addition of exogenous Gas6. A level of readout STAT1 phosphorylation, expressed as a normalized “Activation Index,” greater than 2.0 serves as an objective criterion for functional apoptotic-level phosphatidylserine externalization. The presence of apoptotic cells among viable cells in all cell cultures may account for the modestly elevated activation index of viable cell samples. As reported previously (61), the phosphatidylserine-mediated enhancement of ligand-induced MER activation was greatly reduced in the presence of annexin V (see below; Fig. 5*B*), consistent with the binding of annexin V to phosphatidylserine and the ability of annexin V to thereby act as a competitor of Gas6 for phosphatidylserine-binding sites.

The analysis of phosphatidylserine externalization by viable W3 - I1dm cells and by viable W3 - CDC50A^ED29^ is presented in Figure 3*C*. Whereas, viable W3 - I1dm cells do not trigger Mer activation substantially, W3 - CDC50A^ED29^ cells that lack the ATP11C phospholipid flippase activate Mer strongly. These results, which are entirely consistent with the patterns of phosphatidylserine externalization observed cytofluorimetrically (Fig. 1), extend those data to demonstrate that the phosphatidylserine externalized constitutively by W3 - CDC50A^ED29^ cells is functional, independent of apoptosis. Also consistent with the cytofluorimetric data of Figure 1, apoptotic PLB-985 cells, just like viable PLB-985 cells, fail to externalize phosphatidylserine (Fig. 3*D*; note that this immunoblot was overexposed to reveal the very low level of phosphatidylserine externalization of those cells).

### Apoptotic cells exert ubiquitous immunomodulatory effects independently of externalized phosphatidylserine

The immunomodulatory activity of apoptotic cells reflects the modulation of inflammatory responses primarily on the level of transcription in cells recognizing them (5, 6). The inhibition of proinflammatory cytokine gene expression and induction of anti-inflammatory cytokine gene expression are hallmarks of IAI (5, 6, 12). We examined the abilities of our collection of cells with altered patterns of phosphatidylserine externalization to affect IAI. We assessed NFκB-dependent transcriptional activity and tumor necrosis factor-α (TNFα) secretion as measure of proinflammatory responses and the secretion of interleukin 10 (IL-10) as an anti-inflammatory marker. It is important to reiterate that apoptotic cells are recognized and trigger IAI in a species-unrestricted manner (5, 6) since this collection includes cells of murine and human origin.

**Figure 4:**
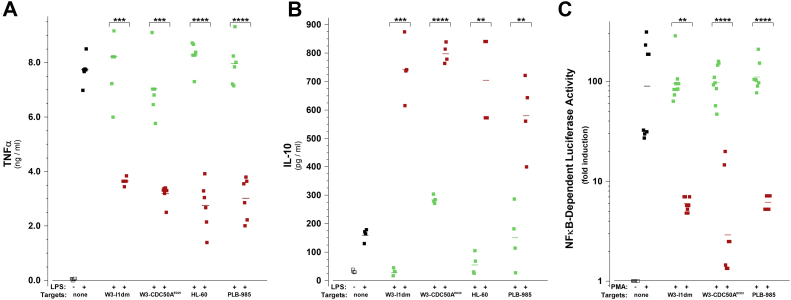
**Apoptotic cells, independent of externalized phosphatidylserine, exert ubiquitous immunomodulatory effects.***A*, TNFα secretion from RAW264.7 macrophages incubated for 6 h without or with the TLR-4 agonist LPS (100 ng/ml) and/or target cells (W3 - I1dm, W3 - CDC50A^ED29^, HL-60, or PLB-985) at T:R = 5:1, as indicated. Target cells were pretreated with staurosporine (“apoptotic”; ) or left untreated (“viable”; ). *B*, IL-10 secretion from RAW264.7 macrophages incubated for 24 h without or with LPS (100 ng/ml) and/or target cells at a ratio of target (T) cells added per responder (R) cell (“T:R ratio”) of 5:1, as indicated. *C*, NFκB-dependent luciferase activity from B2 reporter cells (6) incubated for 18 h without or with phorbol myristate acetate (PMA, 1.25 ng/ml) and/or target cells at T:R = 5:1, as indicated. For (*A*) and (*B*), all conditions were replicated in duplicate cultures and ELISA determinations of each sample also were replicated in duplicate. For (*C*), each condition was repeated in nine replicate wells, and the luciferase activities in cells from each well were determined separately. *Bars* indicate the geometric mean of replicate determinations for each sample. For each target cell line, the significance of differences in TNFα and IL-10 secretion and NFκB-dependent luciferase activity resulting from treatment with viable and apoptotic targets, as calculated by Welch's unequal variances *t* test, are indicated (∗∗*p* ≤ 0.01; ∗∗∗*p* ≤ 0.001; ∗∗∗∗*p* ≤ 0.0001). Panels present data from single experiments, which are each representative of at least three independent experiments. LPS: lipopolysaccharide; TLR4, toll-like receptor 4.

As seen in Figure 4 panel (*A*), the secretion of TNFα by a murine macrophage cell line was stimulated upon the engagement of TLR4 by treatment with bacterial lipopolysaccharide (LPS). Characteristic of IAI that stimulation was inhibited strikingly when those LPS-stimulated macrophages encountered target cells undergoing apoptosis, consistent with the dominant anti-inflammatory effect of apoptotic cells (5, 54). Importantly, this anti-inflammatory effect was limited to apoptotic cells and was independent of phosphatidylserine externalization. W3 - CDC50A^ED29^ cells inhibited TNFα secretion only when they were induced to undergo apoptosis, although they externalize phosphatidylserine constitutively (Fig. 1*B*). PLB-985 cells also inhibited TNFα secretion only when they were induced to undergo apoptosis, although they never externalize phosphatidylserine (Fig. 1*D*).

Complementarily, apoptotic cells enhanced robustly the secretion of IL-10, which was stimulated only slightly upon treatment of macrophages with LPS alone (Fig. 4*B*; (12)). Again, as demonstrated particularly with W3 - CDC50A^ED29^ and PLB-985 cells, this anti-inflammatory effect was limited to cells undergoing apoptosis and was independent of phosphatidylserine externalization.

Finally, we evaluated the effects of the assorted target cells with altered patterns of phosphatidylserine externalization on NFκB-dependent transcriptional activity, assessed in the B2 epithelial cell transcriptional reporter (6) that discloses NFκB-dependent transcriptional activity (Fig. 4*C*). Stimulated proinflammatory transcriptional activity was repressed markedly by cells undergoing apoptosis exclusively and without regard to phosphatidylserine externalization. In this case, the reporter cells were stimulated independently of TLRs or other classical innate immune receptors, consistent with previous characterization of the unconventional, TLR-independent immune signaling associated with IAI (5, 6). Notably, apoptotic cells that do not externalize phosphatidylserine were as effective as apoptotic cells that do externalize phosphatidylserine in triggering responses without interfering with the viability of responding cells.

**Figure 5:**
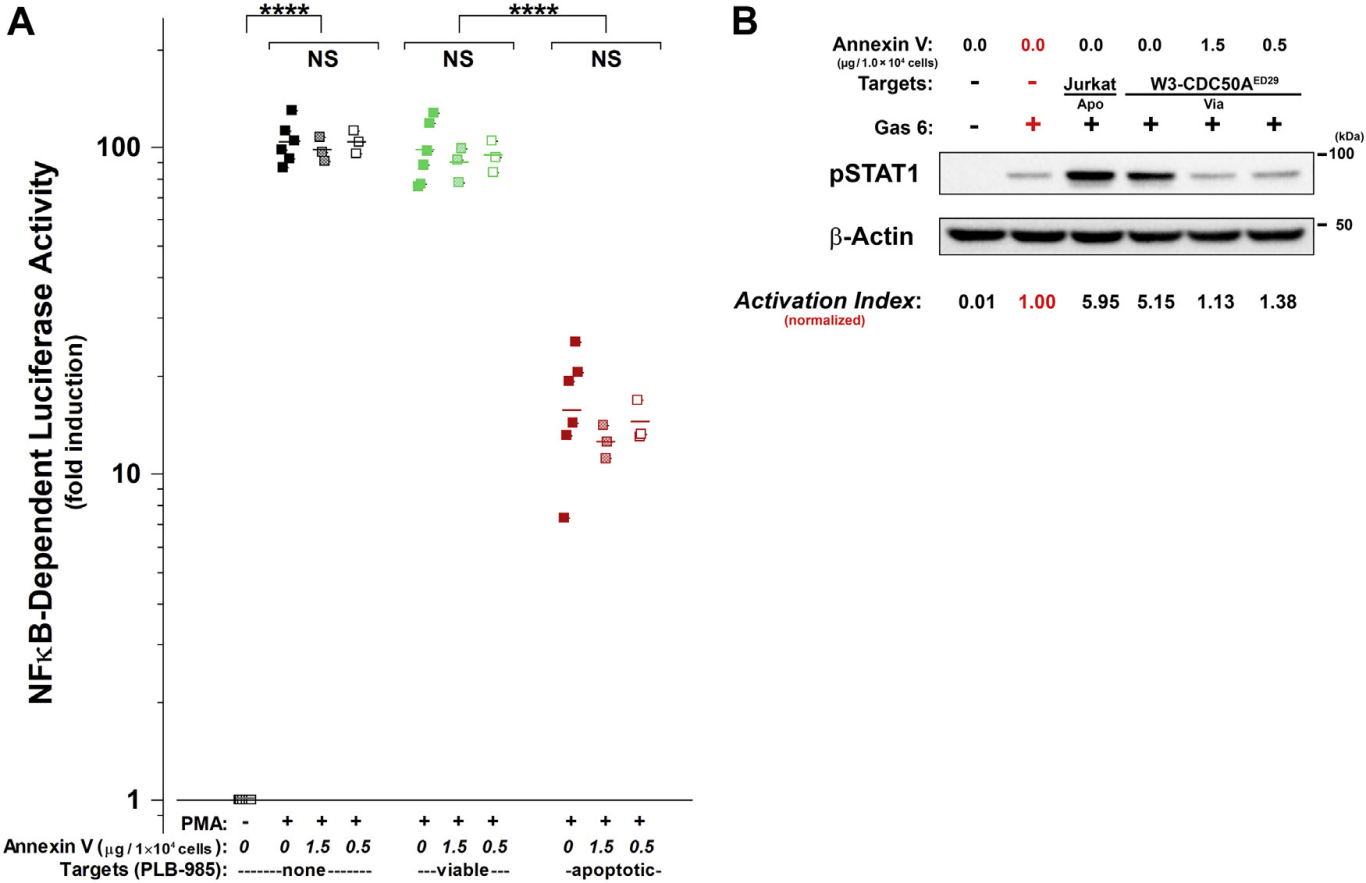
**Apoptotic immunomodulatory effects are unaffected by the masking of phosphatidylserine.***A*, the susceptibility of apoptotic modulation of NFκB-dependent transcriptional activity to the masking of phosphatidylserine by annexin V was assessed as in Figure 4*C*. PLB-985 target cells, which do not externalize detectable levels of phosphatidylserine, were first treated with staurosporine (“apoptotic”; ) or were left untreated (“viable”; ). Unlabeled annexin V then was added to target cells at concentrations that were maximally effective in masking phosphatidylserine (as indicated; see panel *B*) before incubation with B2 reporter cells at T:R = 3:1. Each condition was repeated in replicate wells, and the luciferase activities in cells from each well were determined independently. The significance of differences in NFκB-dependent luciferase activity resulting from treatment with viable and apoptotic targets and as a function of the presence or absence of annexin V, as calculated by Welch's unequal variances *t* test, are indicated (NS: not significant, *p* >0.05; ∗∗∗∗: *p* ≤0.0001). *B*, the phosphatidylserine-masking activity of annexin V was assessed functionally with hMertk-γR1 reporter cells, using STAT1 phosphorylation as the readout, as in Figure 3. Annexin V was added to W3 - CDC50A^ED29^ cells, which constitutively externalize phosphatidylserine (5.0 × 10^5^ W3 - CDC50A^ED29^ cells per lane), as indicated. Panels present data from single experiments, which are representative of at least two independent experiments.

In order to address the possibility that an undetectable level of externalized phosphatidylserine might still be involved, we further tested whether the immune modulation exerted by apoptotic PLB-985 cells is susceptible to annexin V-mediated phosphatidylserine blocking. Analogous to the experiment presented in Figure 4*C*, the immunomodulatory activity of apoptotic PLB-985 cells was evaluated with B2 reporter cells (6). We used a lower ratio of target to reporter cells at which the apoptotic effect was more modest to enhance the sensitivity of detection of possible effects of annexin V blockade. Nonetheless, the repression of proinflammatory NFκB-dependent transcriptional activity exerted by PLB-985 cells when undergoing apoptosis was unaffected by the addition of annexin V, even at saturating concentrations (Fig. 5*A*). The efficacy of annexin V to mask phosphatidylserine is demonstrated by its ability to block the recognition of exposed phosphatidylserine by the chimeric hMertk-γR1 reporter that leads to STAT1 phosphorylation, reducing signal virtually to the phosphatidylserine-independent (and Gas6-dependent) background (Fig. 5*B*).

These data establish that phosphatidylserine is not involved in the triggering of IAI. IAI is exerted specifically by target cells dying apoptotically, independently of phosphatidylserine externalization. Phosphatidylserine is neither sufficient for triggering of IAI, as demonstrated by the absence of immunomodulation by nonapoptotic W3 - CDC50A^ED29^ cells with externalized phosphatidylserine, nor is it necessary, as demonstrated by the IAI response to apoptotic PLB-985 cells lacking externalized phosphatidylserine. The recognition of apoptotic cells that leads to immune modulation occurs independently of phosphatidylserine. Just as phosphatidylserine externalization can be uncoupled from apoptotic cell death, so too can it be uncoupled from apoptotic cell recognition leading to immune modulation.

### Apoptotic cell recognition but not engulfment ensues in the absence of externalized phosphatidylserine

We explored whether we could detect phosphatidylserine-independent recognition of apoptotic cells as the binding of those targets to macrophages. Since recognition leading to phagocytosis is phosphatidylserine dependent (20, 21), we speculated that phosphatidylserine-independent recognition might be revealed especially as the difference between binding without and with ensuing phagocytosis. As in the experiments aforementioned, we employed a cultured macrophage cell line to serve as a homogenous responder population, here for the assessment of binding. We labeled target cells covalently and quantified their interactions with macrophages (54). The phagocytic engulfment of the apoptotic corpse (“efferocytosis”) is dependent on the regulated choreography of actin polymerization (33). Consequently, we evaluated binding in the absence or presence of cytochalasin D, an inhibitor of actin polymerization.

**Figure 6:**
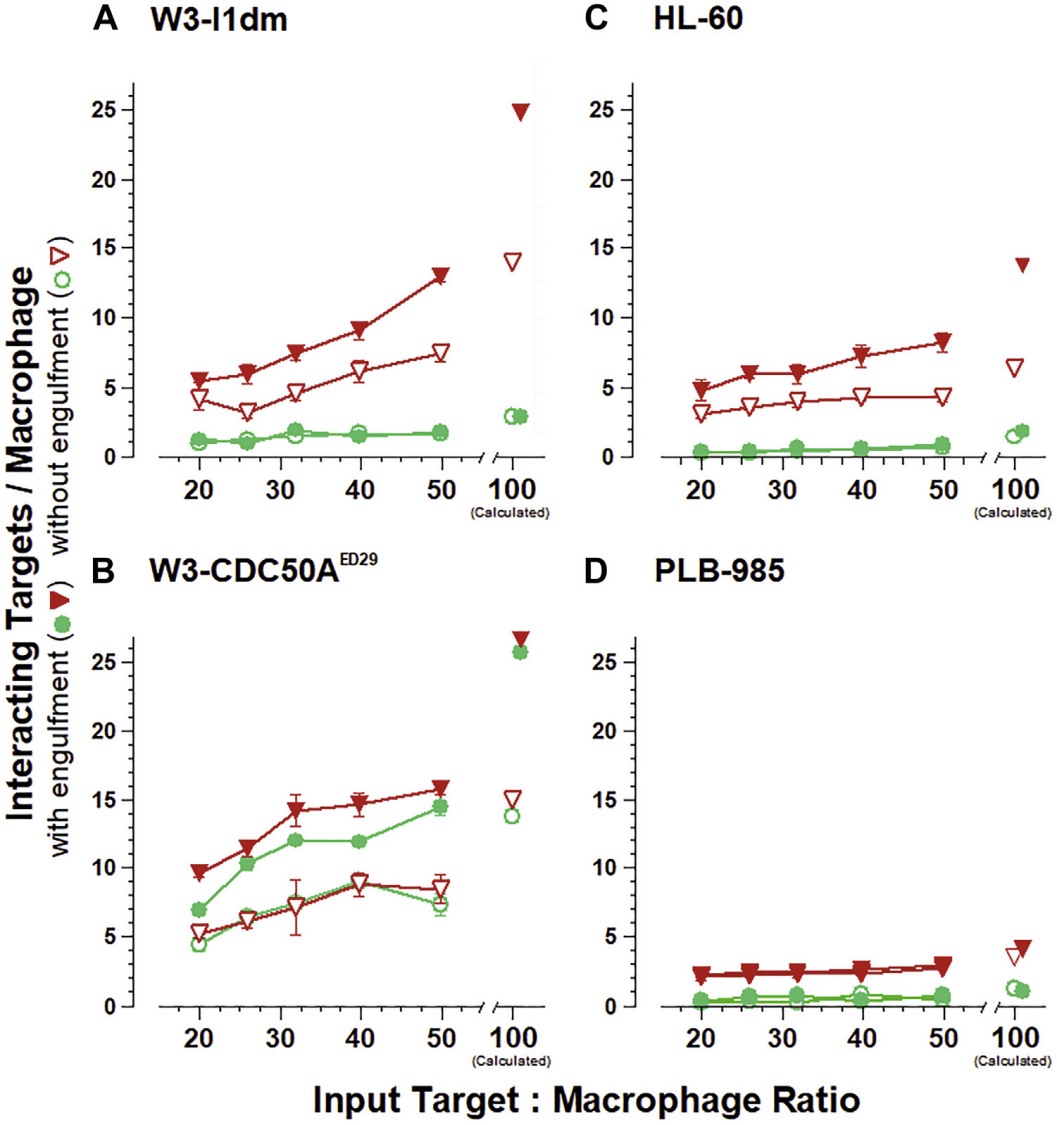
**Apoptotic cell recognition, but not engulfment, ensues independently of phosphatidylserine externalization.** The interaction of target cells with macrophages was quantified as described previously (54). Target cells, covalently labeled with CFDA, were treated with staurosporine to undergo apoptosis (), as in Figure 1, or were left untreated (). Graded numbers of labeled cells were mixed with adherent murine RAW264.7 macrophages in microwells. After incubation for 60 min at 37 °C, unengulfed and unbound cells were removed by washing, and the number of target cells interacting with macrophages () was determined by quantifying CFDA fluorescence (Ex_λ_ = 490 nm; Em_λ_ = 525 nm). Similarly, the extent of interaction without engulfment () was determined in the presence of cytochalasin D (2 μM; (5)). In the absence of macrophages, binding of target cells to microwells was insignificant (*i.e.*, less than the equivalent of 0.1 target cell/macrophage; data not shown). For all panels, error bars represent the SEM of replicate interaction determinations. All conditions were replicated in triplicate wells and fluorescence values for each well were obtained independently. Panels present data from single experiments, which are representative of at least four independent experiments. CFDA, 5(6)-carboxyfluorescein diacetate N-succinimidyl ester.

The interaction of target cells with macrophages (the number of targets bound and/or engulfed per macrophage) is evident as a function of the input ratio of target cells to macrophages (Fig. 6). The interaction of apoptotic W3 - I1dm targets with macrophages (with or without ensuing phagocytosis) was much more extensive than were the interactions of viable W3 - I1dm targets with macrophages (Fig. 6*A*). For purposes of comparison, we extrapolated our determinations of the dose-dependent extents of target cell interactions to target cell:responder cell (T:R) = 100:1 by linear regression. By this approach, it is clear that macrophage interactions with target cells induced to undergo apoptosis (“apoptotic targets”) were at least fivefold greater than were macrophage interactions with untreated target cells (“viable targets”). Here again, the background interaction of cells from untreated cultures may represent interactions with the spontaneously dying cells present in all cell cultures. The interaction of apoptotic targets with macrophages was enhanced when phagocytosis is allowed to ensue. Quantitatively, the extent of apoptotic target cell interactions with macrophages, similarly extrapolated, is enhanced about twofold when phagocytosis ensues.

In contrast, W3 - CDC50A^ED29^ target cells that externalize phosphatidylserine constitutively, whether viable or apoptotic, interacted with macrophages comparably (Fig. 6*B*). This is consistent with the apoptosis-independent phagocytosis of these cells that has been reported previously (23). The almost twofold enhancement of target cell interactions with macrophages when phagocytosis ensued likely reflects this target cell internalization. The magnitude of macrophage interactions with W3 - CDC50A^ED29^ target cells, independent of apoptosis, is similar to that with apoptotic parental W3 - I1dm target cells (compare Fig. 6 panels *A* and *B*). These observations reinforce the view that externalized phosphatidylserine plays a major role in target cell interactions with macrophages. The modest difference in the extents of macrophage interactions between apoptotic and nonapoptotic W3 - CDC50A^ED29^ targets suggests that any role for phosphatidylserine-independent apoptotic determinants in macrophage recognition is minor, relative to the role of externalized phosphatidylserine. Quantitatively, the extent of target cell interactions with macrophages, extrapolated to T:R = 100:1 by linear regression, is enhanced only slightly (1.1-fold) by target cell apoptosis. These data reinforce the view that phosphatidylserine functions as a potent determinant for target cell recognition.

Apoptosis-specific target cell binding was quantifiable with HL-60 cells as well (Fig. 6*C*). The extent of macrophage interactions with HL-60 target cells was less than with W3 - I1dm target cells, likely due to the larger size of HL-60 cells (as distinct from their human origin). Again, as with the WT W3 - I1dm cells, the interactions of apoptotic HL-60 targets with macrophages (with or without phagocytosis) were more extensive than the interactions of viable targets. Quantitatively, the extent of target cell interactions with macrophages without phagocytosis, extrapolated to T:R = 100:1 by linear regression, was enhanced 4.4-fold by target cell apoptosis compared to an apoptotic enhancement of 4.7-fold with W3 - I1dm cells. The magnitude of those interactions was doubled when phagocytosis was allowed to ensue.

Finally, the analysis of PLB-985 cells indicated that, in the absence of externalized phosphatidylserine, the interaction of apoptotic target cells with macrophages can still occur, albeit at much reduced levels (Fig. 6*D*). Apoptotic PLB-985 cells interacted with macrophages more than did untreated PLB-985 cells, but the level of binding was low and cytochalasin D-inhibitable phagocytosis was not detectable. Quantitatively, the extent of target cell interactions with macrophages, extrapolated to T:R = 100:1 by linear regression, was enhanced threefold by target cell apoptosis. However, the extent of macrophage interactions of apoptotic PLB-985 targets was only about half that of apoptotic HL-60 targets. These observations are consistent with the absence of externalized phosphatidylserine on those cells (Fig. 1*D*) and further reinforce the view that externalized phosphatidylserine plays a significant role in target cell interactions with macrophages.

Notably, apoptosis-specific target cell binding, independent of phosphatidylserine, became evident in this phosphatidylserine-negative background. Quantitatively, although the absolute level of target cell binding to macrophages still is modest, the extent of binding was enhanced 2.6-fold with the induction of target cell apoptosis (Fig. 6*D*), compared with only a 1.1-fold increase in the context of constitutively externalized phosphatidylserine (Fig. 6*B*).

### Determinants of apoptotic immunosuppression arise on the apoptotic cell surface independently of phosphatidylserine and phospholipid translocation

**Supplementary Figure 1 Revised:**
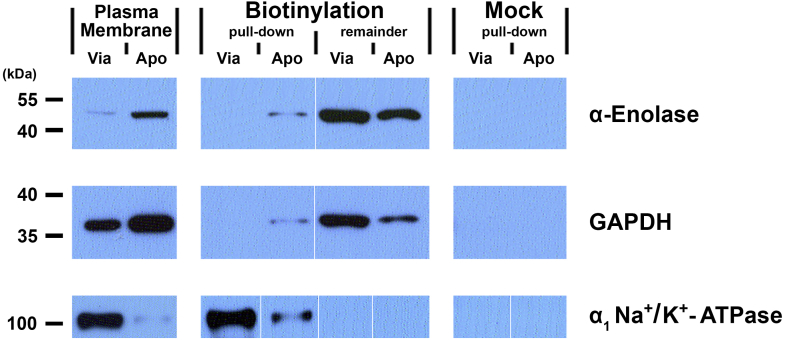


We speculate that this apoptotic target cell recognition by macrophages may be dependent on apoptosis-specific surface-exposed protease-sensitive determinants that we have shown previously to be critical for the recognition of apoptotic targets leading to apoptotic immunomodulation (“SUPER” determinants that are externalized to the apoptotic cell surface, ubiquitously-expressed, protease-sensitive, evolutionarily conserved, and resident generally in viable cells; (1)). Cell fractionation and surface-biotinylation studies with Jurkat cells demonstrate the apoptosis-specific membrane translocation and surface exposure of the SUPER determinants GAPDH and α-enolase (Fig. S1). The observations aforementioned, demonstrating that the recognition of apoptotic cells and the immunomodulation that they trigger occurs independently of phosphatidylserine externalization, suggest that SUPER determinants are externalized and function independently of phosphatidylserine.

**Figure 7:**
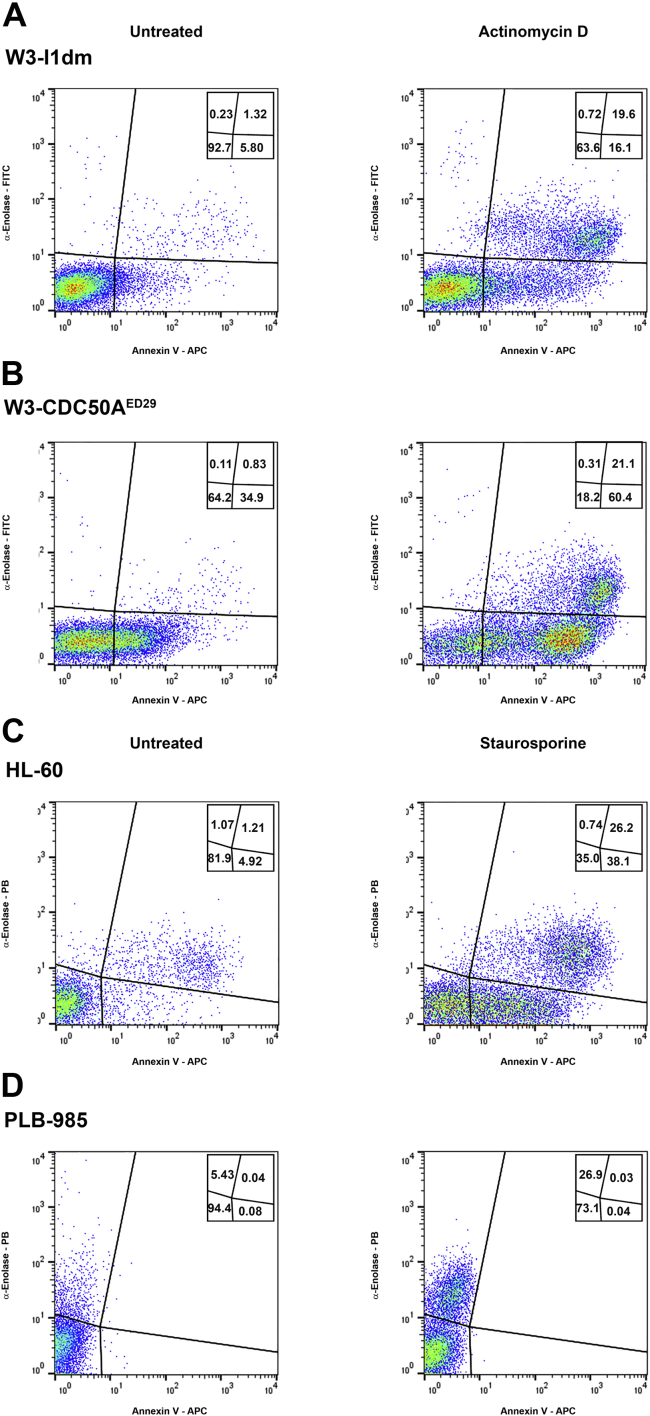
**Protease-sensitive determinants of apoptotic immunosuppression, typified by α-enolase, arise independently of phosphatidylserine translocation.** W3 - I1dm (*A*) and W3 - CDC50A^ED29^ (*B*) cells, untreated or induced to undergo apoptosis with actinomycin D, were analyzed cytofluorimetrically following staining with APC-conjugated annexin V and polyclonal rabbit anti-α-enolase peptide IgG. A fluorescent signal for the α-enolase antibody was developed with a FITC-conjugated secondary anti-rabbit antibody. Quantification of cells within each population, demarcated by relative staining intensity for externalized α-enolase and phosphatidylserine, is indicated. HL-60 (*C*) and PLB-985 (*D*) cells, untreated or induced to undergo apoptosis with staurosporine, were analyzed and quantified similarly, although the secondary antibody used to develop the α-enolase-specific signal was a Pacific Blue-conjugated secondary anti-rabbit antibody. Panels (*A*) and (*B*) and panels (*C*) and (*D*) present data from single experiments, which are representative of more than six independent experiments. APC, allophycocyanin.

We monitored the externalization of the SUPER determinant α-enolase (1). As shown in Figure 7, α-enolase externalization, as assessed cytofluorimetrically, does indeed occur in a strictly apoptosis-specific manner, independently of phosphatidylserine externalization. This apoptosis-specific pattern and the extent (intensity) of α-enolase externalization is unaffected by phosphatidylserine externalization. In particular, whether phosphatidylserine is never externalized (in PLB-985 cells; Fig. 7*D*) or is externalized constitutively (in W3 - CDC50A^ED29^ cells; Fig. 7*B*), α-enolase externalization occurs in an apoptosis-specific manner. As we showed previously, α-enolase externalization is dependent on caspase activity (1). Kinetically, the externalization of phosphatidylserine appears to precede that of α-enolase during the process of apoptosis. The stringently apoptosis-dependent externalization of other SUPER determinants, including GAPDH (see Fig. S2), independent of phosphatidylserine externalization, follows this pattern.

We did note a slight increase in annexin V signal intensity among W3 - I1dm and W3 - CDC50A^ED29^ cells positively stained for surface-exposed α-enolase (Fig. 7 panels *A* and *B*). This is not an artifact of fluorescence spillover from the FITC channel (the fluor with which the α-enolase-specific antibody is tagged in those panels) into the allophycocyanin (APC) (annexin V) channel (*i.e.*, it cannot be “corrected” by compensation). We confirmed both that surface α-enolase-positive cells exhibit elevated annexin V staining and that this minor elevation in the annexin V signal is not subject to compensation by using an α-enolase-specific antibody labeled with a different fluor (Pacific Blue [PB]; see Fig. 7*C*). We observed a similarly increased annexin V signal intensity among surface GAPDH-positive cells (Fig. S2). It may be that the elevated annexin V staining of cells positive for surface expression of α-enolase or GAPDH reflects a modest enhancement of phosphatidylserine accessibility and/or annexin V binding enabled by surface-bound antibody. Since the appearance of SUPER determinants follows phosphatidylserine externalization kinetically, it also is possible that phosphatidylserine externalization has progressed more extensively in those cells.

## Discussion

We have described previously the profound effects on the expression of inflammatory and anti-inflammatory cytokines that are elicited in viable cells upon their recognition of apoptotic cells, which we have termed IAI (5, 6, 7). These effects are exerted independently of apoptotic corpse engulfment and soluble mediators (including TGFβ; (5)). The repertoire of IAI responses is extensive, affecting both the suppression of proinflammatory cytokines and the induction of anti-inflammatory cytokines, and exerted on the level of immediate-early transcription, including NFκB- and AP1-dependent responses (5). In the experiments described in this report, we have assessed the inhibition of NFκB activity, the repression of TNFα expression, and the induction of IL-10 expression (see Fig. 4) as distinct indicators representative of this response repertoire. Previously, we have shown that apoptotic immunomodulation is dependent upon determinants for recognition that are exposed on the apoptotic cell surface in a caspase-dependent manner and that are sensitive to proteolytic digestion (1). Notably, these determinants are conserved and not species specific (6). We now have explored the potential involvement of externalized phosphatidylserine. We have compared IAI responses following interaction with “normal” apoptotic targets and apoptotic targets altered in their externalization of phosphatidylserine.

### Phosphatidylserine-independent triggering of IAI

With this panel of cells, we confirmed that the externalization of phosphatidylserine can be uncoupled from apoptosis. We have documented as well that the externalization of phosphatidylserine can be uncoupled from apoptotic immune modulation. In essence, we have demonstrated both that the triggering of IAI is not impaired in the absence of externalized phosphatidylserine and that the presence of externalized phosphatidylserine itself does not trigger IAI. Previous work has shown that externalized phosphatidylserine triggers phagocytosis even in the absence of apoptosis (23). Our data confirms this as well and further shows that, with regard to apoptotic immunomodulation, the situation is quite the opposite: Target cell triggering of IAI is dependent on the induction of apoptosis and independent of phosphatidylserine externalization.

The behavior of cells that constitutively externalize phosphatidylserine (such as W3 - CDC50A^ED29^) is particularly revealing. Those cells trigger immunosuppressive responses only when they are induced to undergo apoptosis. That is, externalized phosphatidylserine *per se*, although functional and capable of activating the phosphatidylserine receptor Mer and stimulating phagocytosis, is not sufficient to trigger IAI. In addition to addressing the lack of involvement of phosphatidylserine in IAI induction, the failure of nonapoptotic cells with externalized phosphatidylserine to trigger immediate immunomodulatory responses when cultured with Mer-positive macrophages in Gas6-replete medium underscores that the primary immunomodulatory effects that apoptotic corpses exert are Mer independent. Previous studies also have indicated that Mer-dependent immunomodulation is not an immediate-early response (36). The ability of apoptotic cells to trigger anti-inflammatory effects in cells that do not express TAM receptors (6) provides independent support for the conclusion that the contribution to apoptosis-induced immunomodulation arising from phagocytosis-associated TAM activation is a secondary response.

An important unresolved issue is the extent to which phosphatidylserine-dependent effects, including responses elicited upon phagocytosis and by TAM receptors (36, 50), duplicate or supplement phosphatidylserine-independent responses and contribute to the total immunomodulatory repertoire exerted by apoptotic cells. This certainly must include the induction of antigen-specific adaptive immune tolerance (62, 63, 64). While immediate-early phosphatidylserine-independent (and phagocytosis-independent) apoptotic immunomodulatory responses are pleiotropic and long lived (with prolonged responses lasting as long as 24 h as measured *in vitro*; (12)), it may be that they are of particular importance in the establishment of immunomodulation.

### IAI independent of phagocytosis

It is taken as a truism that the purpose of clearance of apoptotic cells is to prevent the release of immunologically provocative intracellular components and ensuing inflammatory responses. The implicit assumption that the immunosuppressive effects elicited by apoptotic cells are linked with the process of engulfment (see (65)) has led to further correlate that deficiencies in clearance lead to deficiencies of immunomodulation. Diminished suppression of immune responsiveness to the autoantigens of which apoptotic cells are a perpetual source might then facilitate the progression of autoimmune pathology. Anecdotal evidence, derived especially from studies of individuals with manifestations of systemic lupus erythematosus (66, 67), has been taken to support this “delayed clearance” model of autoimmune pathogenesis (39, 40). The bases of pathologies associated with delayed clearance have not been established, however. Genetic ablation studies targeting genes involved in apoptotic phagocytosis do not establish a direct link between phagocytosis and inflammation or autoimmunity. For example, it has not been established that pathologies are due to the absence of phagocytosis *per se* or to other signaling functions associated with the altered or absent gene products, as we have noted previously (48, 49). Our previous work (6), which documents the stable persistence of the immunomodulatory activity of apoptotic cells, calls into question the notion that apoptotic cells release DAMPs and become immunogenic and proinflammatory if not engulfed. The results presented here, which demonstrate the dissociation of immunomodulatory ligand exposure from that of the major determinant for phagocytosis, suggest further that the tacit assumption that the immunomodulatory activity of apoptotic cells depends upon their phagocytic clearance and the absence of DAMP release, which is implicit in the “delayed clearance” model, is in need of critical reevaluation.

### IAI determinants for phosphatidylserine-independent recognition

Our results reveal that apoptotic determinants for immunomodulation (“IAI determinants”) appear on the cell surface and function independently of determinants for the phagocytic clearance of those apoptotic corpses, including phosphatidylserine. Together with our previous work, these data suggest that IAI determinants may be comprised exclusively of the conserved protease-sensitive apoptotic cell surface molecules that we identified previously ((1); also see (68)). The externalization of such IAI determinants, for which α-enolase is an exemplar, occurs independently of phosphatidylserine externalization. In fact, apoptotic phosphatidylserine externalization appears normally to precede the externalization of IAI determinants.

Independent of recognition leading to phagocytosis, IAI determinants are molecules for apoptotic recognition leading specifically to target cell binding and immune signaling in responder cells. It is difficult, however, to evaluate apoptotic recognition dependent on IAI determinants (see Fig. 6). While the immunomodulatory responses elicited by IAI determinants are profound (with unambiguous experimental readouts; see Fig. 4), apoptotic recognition dependent on IAI determinants is less readily discerned. This binding is evident only at a low level, apparently masked normally by phosphatidylserine-dependent binding. Apoptotic recognition dependent on IAI determinants was evident in our experiments only with the analysis of cells that did not externalize phosphatidylserine. Clearly, the phosphatidylserine-independent contribution to target cell recognition is dwarfed (and masked experimentally) by the contribution of phosphatidylserine. The exploration of apoptotic cell recognition (binding) in the absence of externalized phosphatidylserine has the unique potential to identify IAI determinants functionally.

### Caspase-dependent triggering of innate apoptotic immunity

Apoptotic cell death in this study was dependent on caspase activity. The importance of caspases in the process of apoptotic cell death, generally considered to be essential, has been a subject of recent reconsideration (8, 52). Caspase involvement in the externalization of phosphatidylserine and, more generally, in the execution phase of cell death notwithstanding, the acquisition of immunomodulatory activity specifically by apoptotic cells is dependent on caspase activity (1, 5). Independently, caspase activity has been shown to play an essential role in the suppression of type I interferon production by apoptotic cells themselves (69, 70).

This requisite function of caspases underscores the irony of the anti-inflammatory effect of apoptosis. While all caspases serve in the sensing of and response to various cellular stresses (due to infection, damage, or other disruption) and initiator caspases rely on proximity-induced mechanisms of autoactivation, some caspases are devoted to promoting inflammation, and others are dedicated to attenuating inflammation *via* the process of immunomodulatory cell death (71, 72). This dichotomy among members of the large caspase family is remarkable. Indeed, the first caspase was identified by virtue of its role in productive processing of proinflammatory cytokines (73). Caspases also have been shown to promote inflammation in other modes of cell death, especially including “immunogenic cell death” (10, 53) and pyroptosis (72, 74). The profound caspase-dependent immunomodulatory effects of apoptotic cells, dissociable from phosphatidylserine externalization and phagocytic recognition, cautions that therapeutic approaches to enhance apoptotic cell death, especially targeting tumor cells, need consider the compromising immunological consequences of apoptosis.

## Experimental procedures

### Reagents

#### Antibodies and fluorescent reagents

Polyclonal rabbit anti-α-enolase and anti-GAPDH antibodies were purchased from Abcam. Mouse monoclonal antibody a6F, specific for the α_1_ subunit of the Na^+^/K^+^-ATPase was obtained from the Developmental Studies Hybridoma Bank at the University of Iowa. Secondary goat anti-rabbit antibodies conjugated with FITC or PB were purchased from Invitrogen/Thermo Fisher. Annexin V coupled to APC was purchased from BioLegend. Unlabeled recombinant human annexin V was from Sino Biologicals (Thermo Fisher). TMRM and 5(6)-carboxyfluorescein diacetate N-succinimidyl ester (CFDA) were purchased from Invitrogen/Thermo Fisher.

#### Other reagents, inhibitors, and drugs

Actinomycin D and staurosporine (from *Streptomyces* sp.), LPS (from *E. coli* 011:B4), and cytochalasin D (from *Zygosporium mansonii*) were purchased from Sigma–Aldrich. The pan-caspase inhibitor Q-VD-OPh was purchased from R&D Systems.

### Cell culture and induction of cell death

All freshly cloned cells were grown at 37 °C in a humidified, 5% (vol/vol) CO_2_ atmosphere in RPMI1640 medium (Mediatech) supplemented with L-glutamine (2 mM), 2-mercaptoethanol (50 μM), and heat-inactivated fetal bovine serum (FBS) (10% vol/vol; Hyclone Laboratories), except PLB-985, HL-60, and B2 cells, which were grown similarly in Dulbecco's modified Eagle's medium (DMEM) with 4.5 gm/liter glucose (Mediatech) supplemented with 10% (vol/vol) FBS and 2 mM L-glutamine, and not with 2-mercaptoethanol, and hMertk-γR1 reporter cells, which were grown in Ham’s F-12 medium (Corning) supplemented with 10% (vol/vol) FBS.

Several target cell lines with altered patterns of phosphatidylserine externalization, as well as the respective parental cell lines, were employed in this study. These include the following:

W3 - CDC50A^ED29^, a murine T cell line that externalizes phosphatidylserine constitutively due to the targeted ablation of the phospholipid flippase chaperone CDC50A (23).

W3 - I1dm, the parental murine transformed T cell line from which W3 - CDC50A^ED29^ is derived. W3 - I1dm itself is derived from the WR-19 Abelson-transformed T cell leukemia.

PLB-985, a human promyelocytic leukemia cell line (75) that fails to externalize phosphatidylserine during apoptosis. The lack of *Xkr8* expression in PLB-985 cells accounts for the absence of apoptosis-associated phosphatidylserine externalization by those cells (27).

HL-60, a human acute promyelocytic leukemia cell line, the parental source of PLB-985 (57); as well as

Jurkat, a human T leukemia cell line, with a WT-like pattern of phosphatidylserine externalization that we have characterized previously (6, 61).

Physiological cell death (apoptosis) of target cells was induced by treatment with the macromolecular synthesis inhibitor actinomycin D (200 ng/ml, 18 h; (58)) or the protein kinase inhibitor staurosporine (1 μM in serum-free medium for 3 h; (12)). Target cells then were washed twice in complete medium before culturing with responder cells. Caspase-dependent apoptosis was inhibited with the pan-caspase inhibitor Q-VD-OPh (10 μM).

A variety of responder cells also were employed. These include the following:

RAW264.7, a murine (H-2^d^) monocyte-derived macrophage cell line (5, 54) B2, a NFκB-dependent transcriptional reporter cell line derived from HEK 293T cells (6); and hMertk-γR1 reporter cell line, a cell line derived from Chinese hamster ovary cells expressing a chimeric Mer reporter construct (61).

In many of the experiments described here, the relative number of target (T) cells added per responder (R) cell is described with respect to the “T:R ratio”.

### Cytofluorimetric analyses

Cell death was assessed cytofluorimetrically by the loss of mitochondrial membrane potential, as indicated by the relative intracellular accumulation of TMRM (Ex_λ_ = 488 nm, Em_λ_ = 585 ± 21 nm; (58)) in cells that retained plasma membrane integrity (*i.e.*, in cells with normal forward- and side-angle light scatter not stained by propidium iodide [PI^-^ cells]).

Phosphatidylserine externalization of [viable] cells not treated to undergo apoptosis and cells treated to undergo apoptosis was assessed cytofluorimetrically (BD LSRFortessa or Attune instruments; BD Biosciences and Thermo Fisher, respectively) by staining with annexin V coupled to APC(Ex_λ_ = 640 nm, Em_λ_ = 665 ± 20 nm) or phycoerythrin (Ex_λ_ = 561 nm, Em_λ_ = 575 ± 25 nm).

The surface exposure of α-enolase and GAPDH also were assessed cytofluorimetrically, simultaneously with annexin V-dependent detection of externalized phosphatidylserine, on unfixed and unpermeabilized cells, using polyclonal rabbit anti-α-enolase peptide and anti-GAPDH antibodies and a fluorescent secondary anti-rabbit antibody conjugated with FITC (Ex_λ_ = 488 nm, Em_λ_ = 530 ± 40 nm) or PB (Ex_λ_ = 405 nm, Em_λ_ = 450 ± 50 nm). Cytofluorimetric data were processed with FlowJo software (BD Biosciences).

### Assessment of phosphatidylserine-dependent signaling

The functionality of externalized phosphatidylserine also was assessed with a reporter assay for phosphatidylserine-dependent pSTAT1 phosphorylation, as described (61). In brief, this assay of externalized phosphatidylserine relies on its detection using a chimeric reporter construct comprised of the human Mer ectodomain and the IFNγR1 transmembrane and cytoplasmic regions, where dimerization resulting from phosphatidylserine-bound Gas6 induces the phosphorylation of STAT1 (see Fig. 3*A*). hMertk-γR1 cells (61) were plated in 6-well plates (1.0 × 10^6^ cells/well), cultured overnight at 37 °C, and serum starved for 6 h. Target cells or large multilamellar phosphatidylserine vesicles (prepared essentially as described; (61)) were added in a final volume of 1 ml complete Ham’s F-12 Medium, including 100 μl of conditioned medium from Gas6-expresing HEK 293T cells (61), as indicated, and cultures were incubated for 30 min at 37 °C. Serum-free DMEM medium (1.8 mM CaCl_2_) was used for experiments involving Ca^2+^-dependent annexin V blocking. Annexin V was added to target cells (at the doses indicated) in medium containing Gas6 before incubation with hMertk-γR1 cells. After washing with Ca^2+^-Mg^2+^–free PBS, whole-cell lysates were prepared in HNTG buffer (50 mM Hepes, pH 7.4; 150 mM NaCl; 10% Triton X-100; 10% glycerol; 1.0 mM Na_2_EDTA; 1.0 mM Na_3_VO_4_; 10 mM Na_2_MoO_4_; 1.0 mM PMSF; and 1% halt protease and phosphatase inhibitor cocktail [Thermo Fisher]). Extract samples (40 μg protein, determined by Bradford assay) were processed using SDS PAGE and blotted. STAT1 phosphorylation ensuing from the phosphatidylserine/Gas6-dependent activation of the Mertk reporter construct was detected with an antibody specific for phosphotyrosine 701 of pSTAT1 (#612232, pY701; BD Biosciences) and normalized to β-actin (detected with antibody #8H10D10; Cell Signaling).

### Assessment of inflammatory response modulation

ELISA analyses were performed to quantify secreted cytokine levels. An NFκB-dependent luciferase reporter was used to evaluate NFκB-dependent activity.

#### TNFα suppression

A total of 2.5 × 10^5^ RAW264.7 macrophages and 1.25 × 10^6^ of the indicated targets (*i.e.*, T:R = 5:1) were cultured in 2 ml/well of a 24-well plate. Secreted TNFα levels in culture supernatants at 6 h were quantified by ELISA (BioLegend ELISA MAX Deluxe Set Mouse TNF-α; #430901), as per the manufacturer’s instructions.

#### IL-10 induction

A total of 2.5 × 10^5^ RAW264.7 macrophages and 1.25 × 10^6^ of the indicated targets (*i.e.*, T:R = 5:1) were cultured in 2 ml/well of a 24-well plate. Secreted IL-10 levels in culture supernatants at 24 h were quantified by ELISA (BioLegend ELISA MAX Standard Set Mouse IL-10; #431411), as per the manufacturer’s instructions.

#### Suppression of NFκB-dependent activity

B2 cells (6) were plated in wells of 24-well plates (1.0 × 10^5^ cells/well) and cultured overnight at 37 °C. The next day, target cells (at a target:B2 cell ratio of 5 : 1) and phorbol 12-myristate 13-acetate (1.25 ng/ml; EMD Biosciences) were added in a final volume of 2 ml as indicated, and cultures were incubated for an additional 18 h. Luciferase activity was determined using the Promega Luciferase Reporter Assay System (E-4030). Cells in each well were lysed (1 × lysis buffer) and analyzed (FB12 luminometer; Zylux) independently. Cell numbers and volumes were reduced for experiments involving annexin V blocking. B2 cells were plated in wells of 96-well plates (1.0 × 10^4^ cells/well), and target cells were added at T:R = 3:1 in a final volume of incubation of 200 μl. Luminescence was assayed in individual wells (EnVision 2103 Multilabel Plate Reader, PerkinElmer) using neolite reporter gene assay system (PerkinElmer).

### Target cell binding to macrophages and phagocytosis

Covalent labeling of target cells and quantitation of target cell interactions with macrophages followed published protocols (5, 54). Briefly, target cells were labeled with the amine-reactive probe CFDA (Thermo Fisher C1157; Ex_λ_ = 485 ± 20 nm; Em_λ_ = 528 ± 20 nm) to a low specific fluorescence intensity (∼4.0 fluorescence units per 1 × 10^2^ cells). Cells (1 × 10^6^ cells/ml in PBS) were incubated with CFDA (2 μM) for 10 min at 37 °C and then washed twice in complete medium. Cells were labeled when viable; for labeled apoptotic targets, CFDA labeling preceded death-inducing treatment. Two hours before the initiation of phagocytosis assays, RAW264.7 macrophages were plated in 96-well flat-bottom tissue culture plates (Costar) at a density of 2.5 × 10^4^ cells/well to allow semiconfluent monolayer formation. Graded numbers of CFDA-labeled target cells were added to the macrophage monolayers, and cells were allowed to interact at 37 °C. In parallel, the interaction of targets with macrophages blocked in phagocytosis was assessed by pretreating macrophages for 45 min with cytochalasin D (2 μM) and continuing incubations with targets in the presence of cytochalasin D. After 60 min of interaction, wells were washed twice with ice-cold PBS, and plate-bound fluorescence was analyzed on a Synergy 2 Fluorescence Plate Reader (Biotek Instruments). For quantitation, a standard curve was prepared with graded number of labeled target cells.

### Cell fractionation and surface biotinylation

Cells were lysed without detergent and fractionated essentially as described (76, 77). Briefly, cells were disrupted by sonication in homogenization buffer (250 mM sucrose, 10 mM Tris, pH 7.4, and 2 mM Na_2_EDTA, and a protease inhibitor cocktail [Roche cOmplete]). Cleared supernatants were fractionated through five-step sucrose gradients. The fraction containing plasma membrane material was isolated at the interface between the 0.8 M and 1.2 M sucrose steps. Note that cell lysis did not involve the high-pH extraction of peripherally associated proteins. Cell-surface biotinylation and protein pull down was accomplished as described (77, 78) with modifications. All procedures involving nonadherent (including apoptotic) cells were performed in suspension, including biotinylation (biotinylation buffer: 150 mM NaCl, 10 mM triethanolamine [pH 7.5], 2 mM CaCI_2_). Cells were lysed with NP-40 (150 mM NaCl, 50 mM Tris HCl [pH 7.5], 1% NP-40), and biotinylated proteins were recovered on Streptavidin-coupled Sepharose beads.

## Data availability

All data have been included within the article and supplementary figures.

## Supporting information

This article contains supporting information.

## Conflict of interest

The authors declare that they have no conflicts of interest with the contents of this article.

## Figures and Tables

**Supplementary Figure 2:**