# Supplementary material for: Phosphatidylserine externalization by apoptotic cells is dispensable for specific recognition leading to innate apoptotic immune responses
Source: J Biol Chem. 2022 May 16;298(7):102034. doi: 10.1016/j.jbc.2022.102034 (PMC9234239; doi:10.1016/j.jbc.2022.102034)
Supplement: Supplementary Figures 1 and 2 Legend [file mmc1.docx]

**Supplementary Figure 1. Apoptosis-specific membrane translocation and surface exposure of the SUPER determinants α-enolase and Glyceraldehyde-3-phosphate dehydrogenase (GAPDH).**

The localization of SUPER determinants was assessed in Jurkat cells (viable [“Via”] and apoptotic [“Apo”] cells induced to die with staurosporine) by fractionation and surface-biotinylation. Fractionated plasma membrane material (“Plasma Membrane”) was separated electrophoretically. The migration of molecular weight size standards is indicated to the left. Separated proteins were blotted and probed with antibodies specific for the SUPER determinants α-enolase and Glyceraldehyde-3-phosphate dehydrogenase (GAPDH), as well as for the α1 subunit of the Na^+^ / K^+^ - ATPase. The presence in this fraction of the Na^+^ / K^+^ - ATPase, an integral plasma membrane protein, serves as a positive control for the procedure. Following cell-surface biotinylation, proteins that bound (“pull-down”) or that did not bind (“remainder”) to Streptavidin beads were separated and probed similarly. Streptavidin-binding proteins also were recovered following a reaction that did not include the reactive succinimidyl ester of biotin (“Mock”). The figure presents data from a single experiment, which is representative of more than eight independent experiments. Here, samples probed for the Na^+^ / K^+^ - ATPase α1 subunit were run on separate gels. In the assembled figure, white borders demarcate gel slices that were rearranged for clarity.

**Supplementary Figure 2. The apoptosis-specific externalization of GAPDH, a SUPER determinant, occurs independently of phosphatidylserine translocation.**

W3 - I1dm (**A**) and W3 - CDC50A^ED29^ (**B**) cells, untreated or induced to undergo apoptosis with actinomycin D, were analyzed cytofluorimetrically following staining with APC-conjugated annexin V and polyclonal rabbit anti GAPDH antibody. A fluorescent signal for the GAPDH-specific antibody was developed with a FITC-conjugated secondary anti-rabbit antibody. Quantification of cells within each population, demarcated by relative staining intensity for externalized GAPDH and phosphatidylserine, is indicated. The figure presents data from a single experiment, which is representative of three independent experiments.
